# Leber Hereditary Optic Neuropathy (LHON) in Patients with Presumed Childhood Monocular Amblyopia

**DOI:** 10.3390/jcm12206669

**Published:** 2023-10-22

**Authors:** Sanja Petrovic Pajic, Ana Fakin, Maja Sustar Habjan, Martina Jarc-Vidmar, Marko Hawlina

**Affiliations:** 1 Eye Hospital, University Medical Centre Ljubljana, Grablovičeva 46, 1000 Ljubljana, Slovenia; dr_sanja_petrovic@hotmail.com (S.P.P.); ana.fakin@gmail.com (A.F.); sustar.majchi@gmail.com (M.S.H.); martina.jarcvidmar@gmail.com (M.J.-V.); 2 Clinic for Eye Diseases, University Clinical Centre of Serbia, 11000 Belgrade, Serbia; 3 Faculty of Medicine, University of Ljubljana, 1000 Ljubljana, Slovenia

**Keywords:** LHON, amblyopia, low visual acuity, retinal thickness, segmentation analysis, visual acuity improvement

## Abstract

Background: Most Leber hereditary optic neuropathy (LHON) cases are bilateral and sequential; however, there are rare unilateral examples, or those in which the delay of onset of vision loss between one and the other eye is longer. In the case of presumed childhood amblyopia in one eye, vision loss in the good eye may be the only symptom of bilateral disease, which was unnoticed in the previously amblyopic eye, or a preexisting episode of LHON in the “amblyopic” eye. The clinical decision in such cases may be difficult and suggestive of other forms of atypical optic neuropathy until confirmed by genetic testing. Case series: We present three genetically confirmed (MT-ND1:m.3700G>A, MT-ND6:m14484 T>C, and MT-ND4:m.11778G>A) patients with subacute vision loss in the previously good eye, with the other eye believed to be amblyopic from childhood and their features different from what would be expected in true amblyopia. In all, electrophysiology testing showed a bilaterally reduced amplitude of PERG with low VEP P100 wave amplitudes and prolonged peak time in both eyes, also unusual for amblyopia. During follow-up, the pallor of the optic discs progressed in all eyes. Significant thinning of the peripapillary retinal nerve fiber layer (pRNFL; retinal nerve fiber layer around the optic disc) and ganglion cell complex (GCC) in the macular region was present. All three patients had a peculiar history. The first patient was treated for presumed hyperopic amblyopia that did not improve since childhood, experienced visual loss in the good eye at the age of 17, and was negative for the three typical LHON mutations. Extended testing confirmed an atypical pathogenic variant MT-ND1:m.3700G>A in homoplasmy. The second patient with presumed strabismic amblyopia had an unusual presentation of vision loss only at the age of 61, and after the exclusion of other causes, a typical MT-ND4:m.11778G>A pathogenic variant was found in homoplasmy. The third case was peculiar as he had presumed strabismic amblyopia since childhood and had some degree of disc pallor in the amblyopic eye upon presenting with loss of vision in the good eye at the age of 21, and a typical pathogenic variant m14484 T>C, p.Met64Val was subsequently confirmed. However, one year after disease onset, he started to experience significant spontaneous functional improvement in the non-amblyopic up to 1.0 Snellen whilst improvement in the presumed amblyopic eye was modest, suggesting preexisting amblyopia. This interestingly extensive improvement was carefully followed by electrophysiology as well as visual acuity and fields. Conclusions: This report shows three different scenarios of presentation of LHON in patients with presumed uniocular amblyopia from childhood. In such cases, the diagnosis may be difficult, and detailed structural and functional evaluation of the optic nerve head is necessary to assess whether an earlier LHON episode was misdiagnosed as amblyopia or whether LHON presented bilaterally on both eyes whilst only being noticed in the previously good eye.

## 1. Introduction

Leber hereditary optic neuropathy (LHON) is a mitochondrial neurodegenerative disease characterized by painless, acute, or subacute loss of the central visual acuity (VA). The disease usually affects both eyes, either simultaneously in 25% of patients or sequentially within a few weeks or months [1]. However, individual unilateral cases [2,3,4,5,6,7,8,9,10,11] and cases with delayed onset between two eyes have also been reported [12]. Childhood LHON is distinct from the adult form of the disease with a better visual prognosis and a more varied clinical presentation, which can be insidious, subclinical, slowly progressive, and in some cases unilateral [4,10,13,14,15,16,17,18,19,20]. The atypical age of onset and clinical patterns of visual loss frequently result in significant diagnostic delays with possible initial misdiagnoses most often as amblyopia. Amblyopia is a visual disorder characterized by a subnormal visual acuity (VA) of different grades in one or both eyes, caused by either visual deprivation or abnormal binocular interactions with normal retinal and optic nerve structures [21].

In this paper, we present detailed phenotypes and a follow-up of three genetically confirmed LHON patients with one eye believed to be amblyopic since childhood that presented with LHON in the good eye later in life.

## 2. Materials and Methods

Three patients who had poor visual acuity in one eye since childhood were selected from the cohort of genetically confirmed LHON patients at the Eye Hospital, University Medical Centre Ljubljana. According to medical history data, low vision in one eye was identified already in childhood, years before the typical presentation of LHON in the other eye. Ophthalmological examinations were performed at the presentation as well as during follow-up periods and included best-corrected visual acuity (Snellen chart, decimal notation), color vision (Ishihara plates), visual field examination (Goldman or Octopus perimetry), fluorescein angiography (FA), and electrophysiology testing. Visual acuities counting fingers and hand motion were expressed as decimal numbers and converted to LogMAR [22,23]. Ring analysis of the retinal nerve fiber layer around the optic disc (peripapillary retinal nerve fiber layer (pRNFL)) was performed with spectral-domain optical coherence tomography (SD-OCT), and segmentation of the retinal layers was performed with the Spectralis HRA apparatus (Heidelberg Engineering, Heidelberg, Germany) in nine Early Treatment Diabetic Retinopathy Study (ETDRS) grid fields [24,25]. Electrophysiological testing with large-field pattern electroretinogram (PERG) was performed, and visual evoked potentials (VEP) were recorded with the Espion visual electrophysiology testing system (Diagnosys LLC, Littleton, MA, USA). All electrophysiological tests followed the standards of the International Society for Clinical Electrophysiology of Vision (ISCEV). The pathological variants in mtDNA were identified with Multiplex Ligation-dependent Probe Amplification (MLPA) and next-generation sequencing of the mtDNA. DNA isolation was performed from peripheral blood according to the manufacturer’s recommendations (DNAeasy Midi Kit, Qiagen, Hilden, Germany). MLPA was performed using SALSA MLPA P125-B1 Mitochondria Probemix Kit (MRC Holland, Amsterdam, The Netherlands) to detect the three primary mutations following the manufacturer’s protocol.

Mitochondrial genome sequencing was performed by a next-generation sequencing method on Ion Torrent PGM (Life Technologies, Carlsbad, CA, USA) on the whole mtDNA isolated from the peripheral blood samples according to the previously described protocol [26,27].

Patients provided written informed consent according to regulations of the University Medical Centre Ljubljana; the use of clinical data was approved by the National Committee for Medical Ethics (No.: 0120-626/2019/5; date: 17 March 2020).

## 3. Results

Presented is a case series (three patients) with LHON and a likely past history of amblyopia during childhood and who later developed LHON in the good eye, whilst the changes in the amblyopic eye were subjectively unnoticed.

### 3.1. Patient 1 (MT-ND1:m.3700G>A)

Patient 1 was a male patient of Slovenian origin, who suffered from a sudden painless VA decline in the left eye (LE) at the age of 17. His detailed medical history and disease progression are presented in Supplementary Table S1. As a 6-year-old child, he had been first seen at the eye hospital due to low vision in one: his right eye (RE: 0.1 cum correctionem (c.c.), LE: 0.7 c.c). Hyperopic glasses (+7.0 Dsph and +6.5 Dsph) were prescribed, and VA improved in both eyes to RE: 0.3 c.c. and LE: 1.0 c.c. Amblyopia treatment with the occlusion of the LE was started, but the vision of the RE did not further improve despite occlusion therapy. However, in the absence of other clinical signs, no additional diagnostic workup was performed at that time.

#### 3.1.1. Disease Onset

The patient was referred to the eye hospital 5 months after the insidious onset of a gradual visual decline in the LE at the age of 17. On admission, the RE VA was counting fingers at 2 m (approx. 0.03, 1.52 LogMAR) and LE counting fingers at 1 m (approx. 0.015 Snellen, 1.82 LogMAR). The patient did not subjectively notice any additional visual loss in the amblyopic eye. His refractometry at that time was RE: + 2.0 Dsph–1.25 Dcyl axis 5 LE: + 1.5 Dsph–1.75 Dcyl axis 180, and his eye movements were normal and painless with no squint.

**Figure 1:**
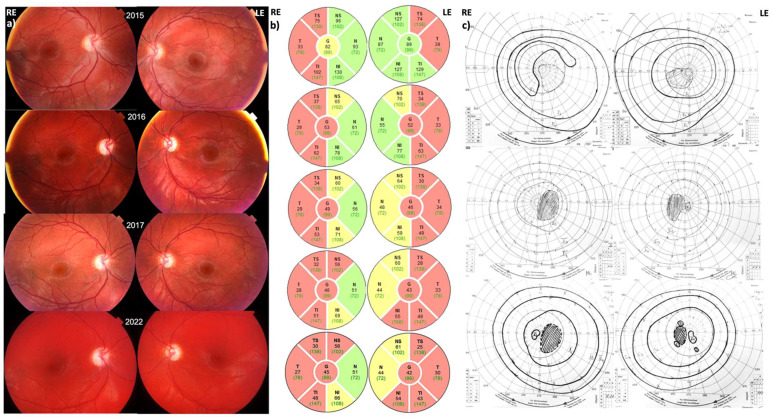
(**a**) Fundoscopy, (**b**) peripapillary RNFL, and (**c**) visual field of Patient 1 at presentation and during the 7-year follow-up period. Note the development of disc pallor in both eyes on the fundoscopy with progressive thinning of pRNFL (marked with red) and persistent central scotoma. Green fields represent parts that still have normal or thickness greater than normal, yellow fields have a borderline thickness, and red fields mark thickness less than normal (atrophy of the peripapillary RNFL).

Color vision was RE 0/15 and LE 1/15, and there was a central scotoma in the visual field of both eyes (Figure 1). Both optic nerve heads (ONH) were mostly pink with some pallor in the temporal part, more in the amblyopic RE, while the blood vessels were slightly tortuous (Figure 1). The thinning of the pRNFL on OCT was present in the temporal half of the optic discs, whilst the nasal was still preserved. Electrophysiology showed reduced amplitudes of PERG N95 waves and reduced amplitude and a prolonged peak time of VEP P100 waves bilaterally, more in the presumed amblyopic RE (Supplementary Table S2). A rare, previously published MT-ND1:m.3700G>A pathogenic variant in homoplasmy was confirmed. His sister and mother were asymptomatic carriers of the same variant.

#### 3.1.2. Follow-Up

During follow-up, his VA did not improve, and disc pallor progressed bilaterally with thinning of the RNFL layer. Segmentation analysis of the retinal layer thickness showed thinning of the GCC in all ETDRS segments in both eyes, which stabilized in the last years of follow-up (Supplementary Table S3). Electrophysiology tests showed the VEP P100 wave was undetectable, and the PERG N95 wave reduced to the level of the baseline in both eyes.

### 3.2. Patient 2 (MT-ND4:m.11778G>A)

Patient 2 was a 61-year-old male of Serbian origin at the time of the painless VA decline in his left, previously normal eye in January 2017. He also did not subjectively notice vision loss in the amblyopic eye. His detailed medical history and disease progression are presented in Supplementary Table S4. He had low vision in his right eye since early childhood, presumably due to a strabismic amblyopia, and he remembered that he had squint surgery.

#### 3.2.1. Disease Onset

**Figure 2:**
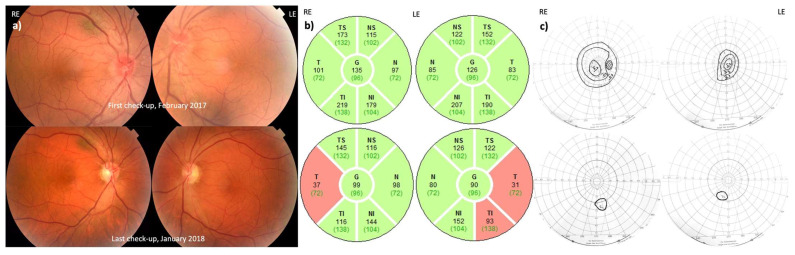
(**a**) Fundoscopy, (**b**) visual field, and (**c**) peripapillary RNFL of Patient 2 at the first visit and at follow-up one year later. Note: pseudoedema of both discs upon onset and temporal pallor and RNFL thinning at the last visit on fundoscopy.

At the time of the examination (two weeks after the visual loss in his previously good LE), the VA was counting fingers at 2 m (approx. 0.03, 1.52 LogMAR) bilaterally, color vision in both eyes was 1/15, and there was a bilateral loss of the entire visual field with some remaining islands of vision seen by searching, with bilateral hyperemic ONH with tortuous blood vessels, typical for the acute stage of LHON. Peripapillary RNFL was swollen and thicker than normal, slightly more in the temporal region of the amblyopic RE in comparison to the LE (Figure 2). Fluorescein angiography showed no leakage. His refraction was RE:+ 2.0 Dsph+ 0.75 Dcyl axis160; LE:+ 2.0 Dsph. His eye movements were normal and painless, without squint.

#### 3.2.2. Follow-Up

Six months after the loss of vision in the LE, electrophysiology showed a decreased amplitude of the PERG N95 and undetectable VEP P100 in both eyes (Supplementary Table S2). Thinning of the pRNFL in the temporal region in both eyes was seen, more prominent in the previously good left eye. Nine months after onset, there was still preservation of the pRNFL in the presumably amblyopic eye in the N, NS, NI, and TS regions, whilst in the LE, the pRNFL was preserved only in the N and NI segments. One year after onset, complete atrophy of the pRNFL was present in both eyes (Figure 2). Thinning of the ganglion cell complex (GCC) was also seen in the segmentation analysis of both eyes (Supplementary Table S3). The pathogenic variant MT-ND4:m.11778G>A, homoplasmy, was then confirmed in the patient. Based on family history, the patient’s mother also suffered from bilateral VA loss at an older age due to optic neuropathy. This patient moved out of the country and was later lost to follow-up.

### 3.3. Patient 3 (m14484 T>C, p.Met64Val)

Patient 3 was a 21-year-old male of Albanian origin from Kosovo who noted gradual painless loss of visual acuity in his RE over a period of 2–3 weeks. He had poor acuity in the left eye since childhood presumed due to amblyopia with severe convergent strabismus (Supplementary Table S5). Two uncles on the mother’s side of the family apparently had a similar episode of vision loss and then improvement at a younger age.

#### 3.3.1. Disease Onset

**Figure 3:**
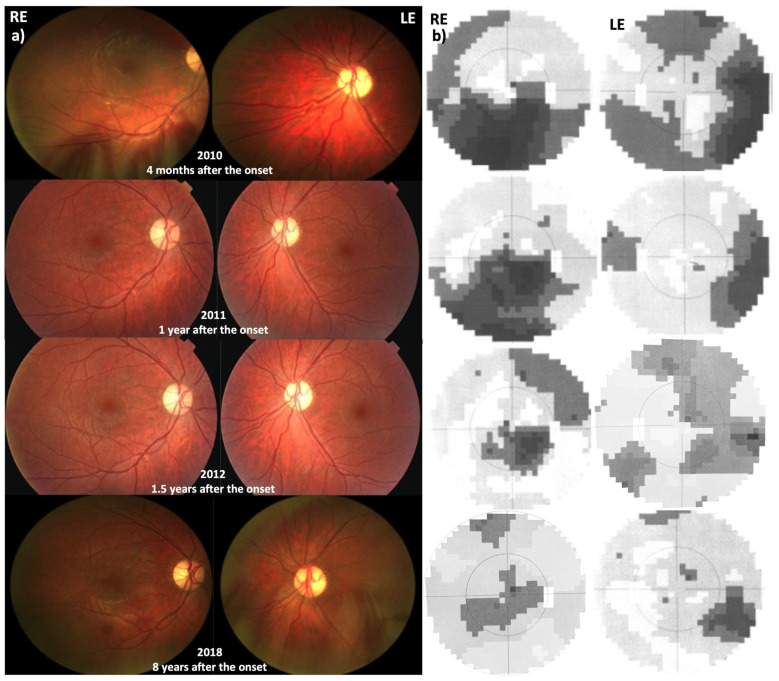
(**a**) Fundoscopy results during follow-up with the pallor of the optic discs. (**b**) Visual fields during follow-up. Note: gradual improvement of the visual field over time despite paler discs.

On admission, three weeks after onset, VA in the previously normal RE was counting fingers at 3 m (approx. 0.05 decimal, 1.30 LogMAR), while in the left, presumably amblyopic eye, it was counting fingers at 2.5 m (approx. 0.04 decimal, 1.40 LogMAR). Loss of vision was subjectively noticed only in the previously good right eye. Color vision was 1/15 in both eyes. Static perimetry showed central and inferior scotoma in the LE and small central scotoma with additional concentric loss of sensitivity in the LE (Figure 3). Both discs were temporally paler, more in the amblyopic eye, with no signs of acute hyperemia.

There was no leakage on fluorescein angiography in either eye. The Pattern ERG N95 wave was decreased, and VEP P100 was decreased and delayed from both eyes, more in the amblyopic eye.

Genetic testing confirmed the typical MT-ND6 pathogenic variant: m14484 T>C, p.Met64Val (homoplasmy).

#### 3.3.2. Follow-Up

**Figure 4:**
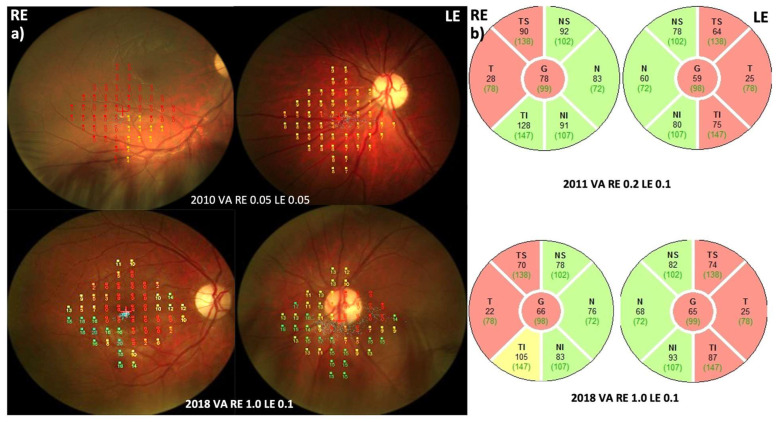
(**a**) Microperimetry and (**b**) pRNFL in Patient 3 at onset and during the follow-up period. Note: good functional improvement in the right eye and, to some extent, in the left amblyopic eye by microperimetry. Despite functional improvement, the thickness of the pRNFL continued to decline slightly during the follow-up period.

Four months after onset, VA was bilaterally 0.1 decimal (1.0 LogMAR) and Ishihara 1/15. Microperimetry, performed 6 months after onset, showed reduced central sensitivity in the RE and extremely eccentric fixation on presumably amblyopic LE (Figure 4). One year after onset, bilateral VA was still 0.1 decimal, Ishihara still 1/15, and discs were paler than earlier in both eyes (Figure 3).

Interestingly, one and a half years after disease onset, VA started to improve (RE 0.2, LE 0.1) and continued to improve to reach 0.8 in the RE two years after disease onset. Color vision improved slightly from 1/15 to 3/15.

Six years later, eight years after onset, at the age of 29, the RE VA improved even to 1.0 Snellen, and color vision improved to 6/15, with fenestration of visual field scotoma and improvement of light sensitivity on microperimetry. VA and color vision in the amblyopic LE did not improve substantially (0.1 Snellen, 1/15 Ishihara). A slight improvement was noted on microperimetry also in the amblyopic eye but was not subjectively perceived (Figure 4).

### 3.4. Segmentation Analysis and pRNFL

In all eyes of Patients 1–3, segmentation analysis of retinal layers showed significant thinning of the GCC in all other segments (Supplementary Table S3) with discrete preservation of the GCC in the central ETDRS field. The amblyopic eyes had an overall thicker retina and GCC in the central ETDRS circle compared to the subacutely affected eyes. No differences were observed in other ETDRS rings or for other retinal layers between the amblyopic and subacutely affected eyes (Supplementary Figure S1). The peripapillary RNFL showed a continued trend of atrophy in both the presumed amblyopic eye and the previously good eye (Supplementary Figures S2–S4), both in the nasal and temporal segments. In Patient 3, who has improved in the good eye, the pattern of GCC thinning showed a progressive decline at disease onset and later stabilization during follow-up in both the good and presumably amblyopic eye (Supplementary Table S3). Improvement of visual acuity was not associated with thickness change.

## 4. Discussion

We describe three patients with monocular low vision in one eye since early childhood, believed to be due to amblyopia, that presented many years after with subacute vision loss in the previously good eye. The question arises whether low vision in the first eye was actually due to an early LHON episode, or was there indeed true amblyopia at that time, with superimposed LHON affecting both eyes later in life, especially as none of the patients reported any loss or change of vision in their amblyopic eye. In the presented patients, unfortunately, no report on optic disc pallor was available in their childhood notes and no OCT or other imaging was available at that time. Patients 1 and 3 already had some pallor of the ONH in the amblyopic eye at the time of the LHON episode in the previously healthy eye. This is an unusual finding in amblyopia and may suggest an earlier episode of LHON. Their different scenarios were therefore clinically challenging and was the reason for the detailed morphological and functional follow-up described in this paper.

Patient 1 lost vision in the good eye at the age of 17, and there was no strabismus, anisometropia or media opacities, or other amblyogenic factors present. Testing for three common genetic pathological variants of LHON was negative. Extended mitochondrial genome testing revealed a rare, previously published [28] MT-ND1:m.3700G>A pathogenic variant in homoplasmy. As on presentation the partial disc pallor was already present in the amblyopic eye, it may be possible that LHON affected the amblyopic eye earlier. However, it is not possible to determine whether LHON was already the cause of “amblyopia” with insidious onset in childhood or whether the true amblyopic eye was additionally affected in a subacute fashion later that remained unnoticed due to poor vision until the good eye was affected. As he had a slight progression of pRNFL thinning during the disease course and electrophysiology was typical for LHON in both eyes, he may correspond to bilateral sequential onset that had initially started in the amblyopic eye; however, is also possible that it could have been caused by a childhood-onset LHON since the patient did not respond to standard amblyopia treatment.

Three different patterns of visual acuity loss were described in childhood LHON: classical acute in 63%, slowly progressive in 15%, and insidious or subclinical in 22% [10]. A recent paper by Barboni et al. [11] proposed a different classification of childhood-onset LHON: subacute bilateral (66.7%), insidious bilateral (17.3%), unilateral (11.1%), and subclinical bilateral (4.9%). According to these studies, different patterns could affect either eye. The specific pattern of insidious unilateral onset in early infancy is quite peculiar, and asymmetric involvement may arise as a consequence of the subtle anatomical differences between the eyes, such as differences in the architecture and size of the optic nerve head, and the number of axons that are known to vary by up to 20% between the eyes [29,30]. In our patient, OCT showed some advancement of atrophy during follow-up also in the amblyopic eye, which could suggest insidious onset and slowly progressive pattern.

Patient 2 showed characteristic ONH hyperemia, tortuosity of blood vessels, and pseudoedema simultaneously in both eyes, although no additional worsening of vision was noted in the previously amblyopic eye. This patient remembered strabismus management with surgery in childhood, in keeping with true strabismic amblyopia. Clinically, this patient had a bilateral symmetric onset of LHON at the rather advanced age of 61, and symmetrical pseudoedema with atypical visual field loss prompted diagnostics towards other neurological causes until genetic testing confirmed the diagnosis.

In Patient 3, the affected eye developed convergent strabismus and could therefore be associated with strabismic amblyopia; however, strabismus can also develop secondary to visual loss due to LHON or any other cause [31]. Partial ONH atrophy was probably present before the second eye was affected since changes in the visual field and color vision were already noted at the first presentation in the absence of acute signs of disease. It is possible that the first attack could have occurred before the age of five (convergent strabismus usually occurs if the visual acuity in one eye is low before the age of five, while divergent strabismus develops at a later age) [31].

In the case of Patient 3, the most probable scenario is insidious unilateral LHON with consequential development of strabismus. Strabismus has been reported in cases of unilateral childhood LHON and is usually associated with low visual function [4,12]. Barboni et al. reported strabismus in all patients with unilateral LHON [11]. They also described a subgroup of patients with the involvement of the second eye at a later age (≥15 years old), which could correspond to our Patient 1. In the insidious unilateral patient group, the younger presumed age of onset and the presence of strabismus could influence the final visual outcome due to a mechanism of cerebral suppression [32,33].

Electrophysiology was characteristic for LHON in both eyes of all three patients, confirming an LHON event at some point. If the fixation is normal, the PERG N95 in amblyopic patients is usually normal [34], whilst in all three patients, the PERG N95 wave was abnormal to a similar extent. In all patients, VEP was also abnormal in both presumably amblyopic and LHON eyes. This confirms that the disease in the presumably amblyopic eye happened prior to or at disease onset in the LHON eye.

All our patients also had low color vision both in the LHON and presumably amblyopic eyes. In the patient with VA improvement, color vision in the presumably amblyopic eye also did not improve. This corroborates with the LHON event in both eyes as color vision is not significantly reduced in amblyopia [35], with some alteration in color perception [36].

With regard to OCT characteristics, either no differences were reported in pRNFL and macular thickness between amblyopic and non-amblyopic eyes [37], or a significantly thicker RNFL was reported in amblyopic eyes [38]. Moreover, a thicker retina in the ETDRS center, thinner in the inner and outer ETDRS ring, was observed in amblyopic compared to normal eyes [39]. Although in our case series, all eyes were affected, and this comparison is not valid, we also noted a thicker GCC in the ETDRS center in amblyopic eyes than in subacutely affected eyes (Supplementary Figure S1 and Supplementary Table S5). Also, all eyes showed a pattern of better preserved GCC thickness in the central ETDRS field than paracentral ones, as described recently, suggesting that both eyes were also affected by LHON [40].

Taking into account everything above, it is most likely that Patients 1 and 3 in this case series suffered from an LHON episode in the presumed amblyopic eye earlier in life but had only noticed unilateral visual loss upon visual loss in the other eye. In Patient 2, the clinical picture was suggestive of severe bilateral subacute LHON, although noted only in the previously good eye.

Delayed involvement of the second eye has been described as rare in adult-onset LHON [12]. Despite the fact that 97% of LHON patients suffer from the involvement of the other eye within one year [2,4], there are a few reports of unilateral or delayed presentation of LHON. Two studies document unilateral vision loss with subsequent visual recovery in the affected eye [3,4]. Several reports describe a delay in fellow eye involvement for a 3–8-year follow-up period [5,6]. Other reports document cases of unilateral involvement with a follow-up period of 18 months [7], 10 years [8], and 16 years [9]. This time difference between the involvement of the two eyes could be explained by the possible presence of a larger number of mutated mtDNA in one optic nerve or the fact that mitochondrial expression in the other eye is increased after the attack in the first eye, for compensating the energy defect [12].

## 5. Conclusions

When faced with acute loss of vision in the good eye associated with presumed amblyopia in the other eye, especially in the presence of optic atrophy or disc swelling in that eye, we should not rule out the possibility of LHON that occurred either in early childhood as the cause of low vision or was superimposed onto true amblyopia later in life and only became manifest upon the involvement of the good eye.

This report shows the importance of detailed structural ONH evaluation with OCT, visual field, and color vision in any child with amblyopia not responding to treatment. It also raises awareness of childhood-onset LHON and the importance of genetic testing in treatment-resistant childhood amblyopia cases.

## Data Availability

The data presented in this study are available on request from the corresponding author.

## Supplementary Materials

The following supporting information can be downloaded at: https://www.mdpi.com/article/10.3390/jcm12206669/s1, Figure S1. Retinal and GCC (ganglion cell complex) thickness in central ETDRS circle in presumably amblyopic and LHON eyes; Figure S2. Peripapillary RNFL changes in the nasal and temporal half of the optic disc in Patient 1; Figure S3. Peripapillary RNFL changes in the nasal and temporal half of the optic disc in Patient 2; Figure S4. Peripapillary RNFL changes in the nasal and temporal half of the optic disc in Patient 3; Table S1. Timeline of the disease progression in Patient 1; Table S2. Electrophysiology data for three patients in both eyes (LHON and presumably amblyopic) in the acute (three patients) and chronic (two patients) phase of the disease; Table S3 Difference in retinal thickness in presumably amblyopic and LHON eye in center, middle, and outer ETDRS ring for all patients; Table S4. Timeline of the disease progression in Patient 2; Table S5. Timeline of the disease progression in Patient 3.
