# Supplementary material for: Leber Hereditary Optic Neuropathy (LHON) in Patients with Presumed Childhood Monocular Amblyopia
Source: J Clin Med. 2023 Oct 22;12(20):6669. doi: 10.3390/jcm12206669 (PMC10607696; doi:10.3390/jcm12206669)
Supplement: Supplementary file 1 [file jcm-12-06669-s001.zip › jcm-2666134-supplementary.pdf]

Journal of
Clinical Medicine

MDPI

Supplementary Materials

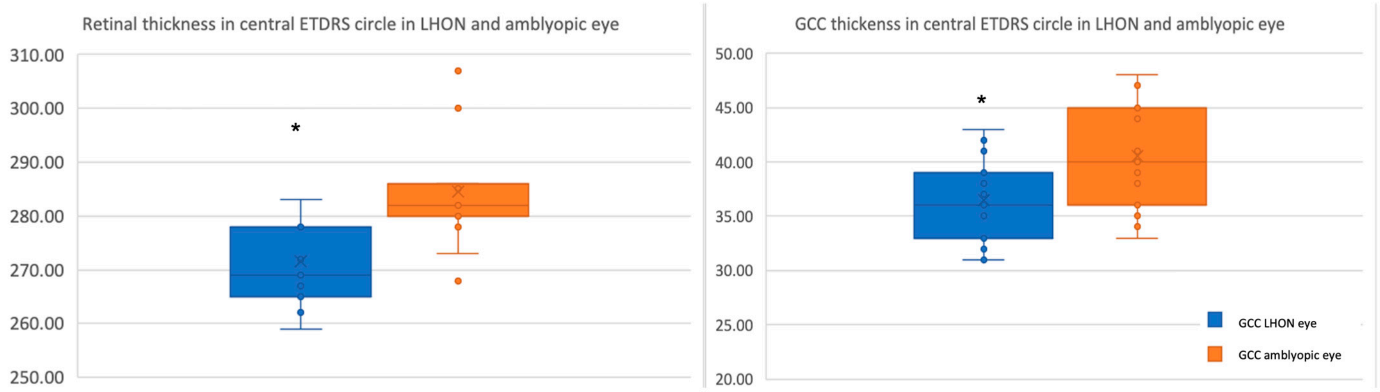

Figure S1. Retinal and GCC thickness in central ETDRS circle in presumably amblyopic and LHON eyes.

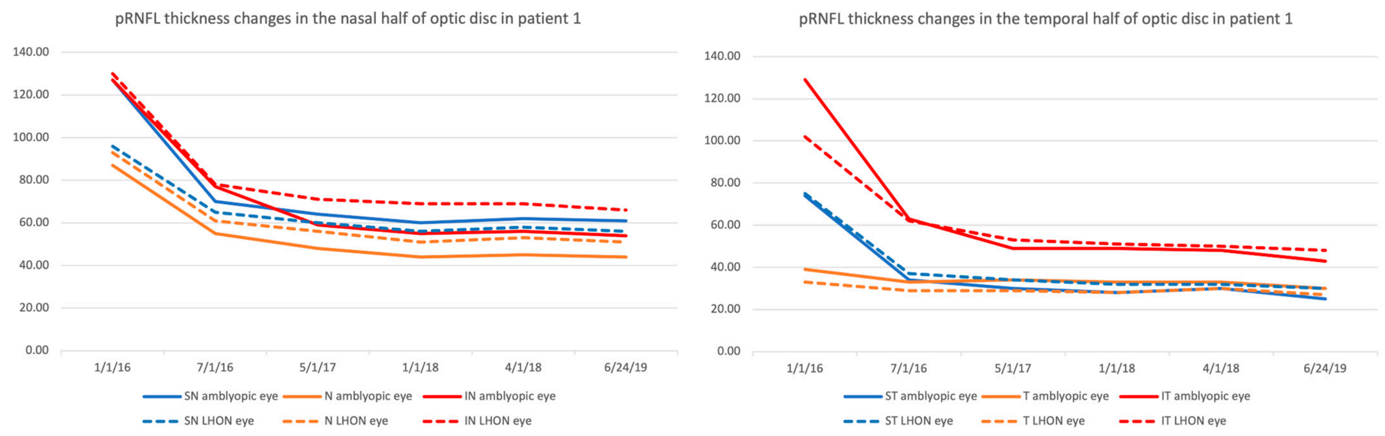

Figure S2. Peripapillary RNFL changes in the nasal and temporal half of the optic disc in patient 1.

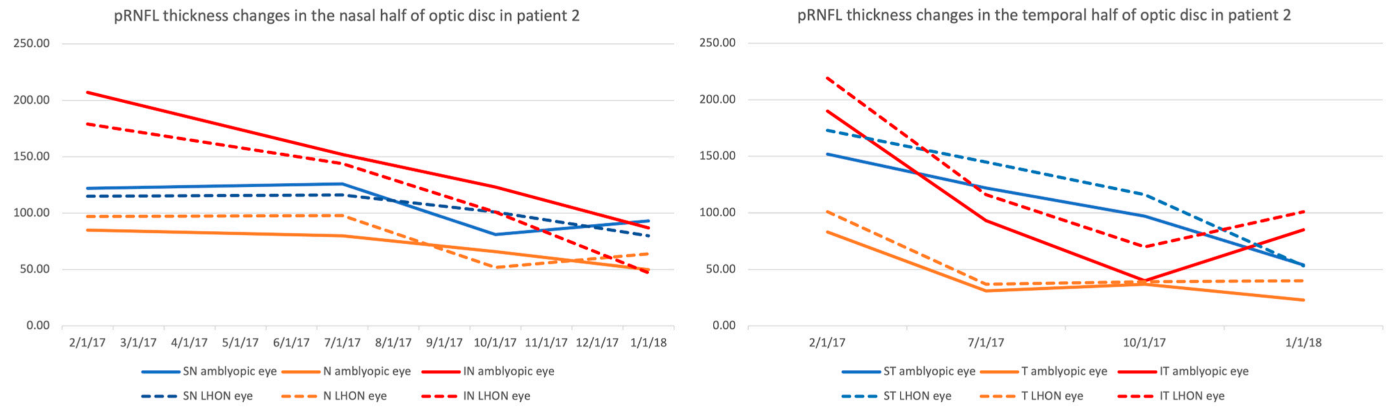

Figure S3. Peripapillary RNFL changes in the nasal and temporal half of the optic disc in patient 2.

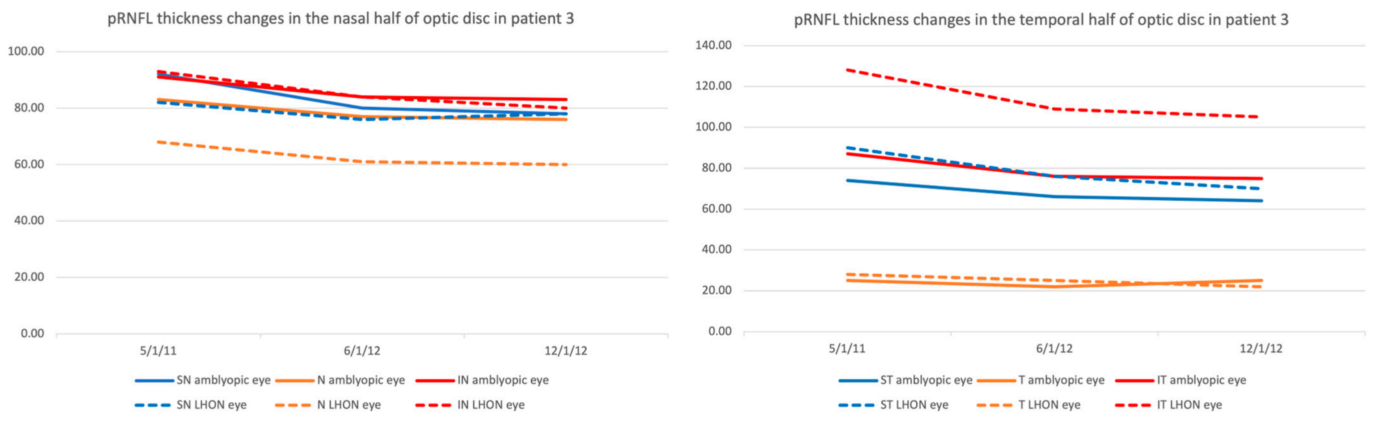

Figure S4. Peripapillary RNFL changes in the nasal and temporal half of the optic disc in patient 3.

Table S1. Timeline of the disease progression in Patient 1.

Date	Relevant past medical history	
	**Patient 1 had low vision in the right eye(RE) since early childhood, presumed to be due to amblyopia, and did not notice any change in visual function in that eye. As a 6 years old child, he had been first seen at the Eye hospital due to low vision in one eye (RE: 0.1c.c, LE:0.7c.c). Hypermetropic glasses (+7Dsph and+6.5Dsph) were prescribed and VA improved on both eyes to RE: 0.3 c.c. and LE: 1.0 c.c. Amblyopia treatment with the occlusion of the LE was started, but the vision of the RE did not further improve despite occlusion therapy**	
	**Summaries from initial and follow-up visits**	**Interventions and therapy**
August 2015	VA loss on the left eye (LE)	Glasses prescription from the local ophthalmologist.
December 2015 (5 months after the onset of the right eye)	Hospitalization at Clinic due to VA loss on the previously healthy left eye. VA RE counting fingers on 2 meters, LE: counting fingers at 1 m Color vision RE 0/15, LE 1/15, central scotoma bilaterally. On fundoscopy reddish optic discs with initial pallor temporally. Thinning of the pRNFL, thinning of the inner retina in the macular region. FA: no leakage, EF: P 50 amplitude normal, lower on amblyopic eye, decreased N95 wave more on the amblyopic eye, N95/P50 ratio RE 0.71 LE:0.78, delayed and decreased VEP P100 with lower amplitude and shorter latency on amblyopic eye.	Systemic corticosteroid therapy for 3 days Solumedrol i.v. (1g/day), no improvement. MRI of the head and brain, no signs of demyelination, aqp4 negative, antiMOG negative, excluded all other possible signs of theoptic atrophy (infectious, paraneoplastic, compressive etc) Blood taken for genetic testing.
July 2016 (one year after the onset)	VA RE counting fingers on 1,5 meters, LE counting fingers at 1 m Color vision RE 0/15, LE 1/15, central scotoma bilaterally. On fundoscopy both optic discs pale, thinning of the inner retina on OCT bilaterally, further progression of the pRNFL thinning	Genetic testing for 3 common mutations negative, mtDNK ngs performed revealed previously published rare mutation, mother and sister tested for the same mutation. Patient started with Idebenone
November 2017 (14 months after the onset on the left eye)	VA RE counting fingers on 1,5 meters, LE counting fingers at 1 m Color vision RE 0/15, LE 1/15, central scotoma bilaterally. On fundoscopy both optic discs pale, thinning of the inner retina on OCT bilaterally, further progression of the pRNFL thinning. EF: P 50 amplitude decreased, more on the amblyopic eye, although still within normal limits, decreased N95 wave on the amblyopic eye, normal on LHON, N95/P50 RE 0.77 LE 1.02, and delayed and decreased VEP P100.	Mutation confirmed in both mother and sister who are asymptomatic carriers
January 2018 (3.5 years after the onset)	VA RE counting fingers on 2,5 meters, LE counting fingers at 2,5 m Color vision RE 0/15, LE 1/15, central scotoma bilaterally. On fundoscopy both optic discs pale, thinning of the inner retina on OCT bilaterally, further progression of the pRNFL thinning. EF: SFERG P50 amplitude improved, more on the amblyopic eye, borderline reduced N95 amplitude improved but the wave was in	

	the level of the baseline, again more on the amblyopic eye<br>N95/P50 RE 0.81 LE 0,92 and delayed and decreased VEP P100.
January 2022 (5.5 years after the on-set)	VA RE counting fingers on 1 meter, LE counting fingers at 0.75 m Color vision RE 0/15, LE 1/15, central scotoma bilaterally. On fundoscopy both optic discs pale, thinning of the inner retina on OCT bilaterally, further progression of the pRNFL thinning, with still preserved nasal segment on both eyes

Table S2. Electrophysiology data for three patients on both eyes (LHON and presumably amblyopic) in the acute (three patients) and chronic (two patients) phase of the disease.

	**Patient 1**				**Patient 2**		**Patient 3**			
	**LHON eye**		**Amblyopic eye**		**LHON eye**	**Amblyopic eye**	**LHON eye**		**Amblyopic eye**	
	Acute	Chronic	Acute	Chronic	Acute	Acute	Acute	Chronic	Acute	Chronic
N95/P50	0.78	0.92	0.71	0.81	0.89	0.76	1.52	1.04	1.27	1.05
Lat P50	45.0	48.0	45.0	47.0	47.0	47.0	40.00	47.0	46.0	45.0
Amp P50	5.4	5.2	4.0	5.8	4.4	3.3	2.9	5.0	2.2	4.0
Lat N95	106.0	94.0	100.0	98.0	106.0	115.0	103.0	95.0	111.0	100.0
Amp N95	4.2	4.8	4.2	4.7	3.9	2.5	4.4	5.2	2.8	4.2
Lat P100	136.0	undetectable	113.0	undetectable	undetectable	undetectable	148.0	110.0	151.0	113.0
Amp P100	1.7	0.0	1.3	0.0	0.0	0.0	1.0	1.4	0.9	1.3

Table S3. Supplementary Table S3 Difference in retinal thickness in presumably amblyopic and LHON eye in center, middle, and outer ETDRS ring for all patients.

			**Retinal thickness**	**GCC thickness**	**INL thickness**	**OPL thickness**	**ONL thickness**	**RPE thickness**
Center	LHON eye	Average	272.21	36.47	27.21	26.14	90.36	17.50
		SD	10.84	3.80	7.68	6.68	13.54	2.31
	Amblyopic eye	Average	284.47	40.53	32.40	26.53	91.73	16.67
		SD	10.51	4.87	7.63	3.021	15.89	1.35
		P value	**0.005**	**0.02**	0.079	0.839	0.801	0.242
Midle ETDRS ring	LHON eye	Average	301.04	63.06	46.30	34.05	74.93	15.96
		SD	24.71	4.70	12.53	8.66	13.33	1.54
	Amblyopic eye	Average	300.48	61.92	43.33	32.69	78.12	15.81
		SD	24.37	5.93	10.31	6.85	14.07	1.54
		P value	0.900	0.879	0.168	0.352	0.217	0.594
Outer ETDRS ring	LHON eye	Average	276.35	65,7	37.79	31.04	60.88	14.53
		SD	17.24	5.65	5.36	3.97	9.13	1.06
	Amblyopic eye	Average	277.33	64,7	38.74	32.59	60.67	14.81
		SD	14.75	5.86	2.82	4.98	9.86	2.18
		P value	0.803	0.92	0.397	0.141	0.926	0.447

Table S4. Timeline of the disease progression in Patient 2.

**Date**	**Relevant past medical history**	
	**Low vision on the right eye since childhood, mother blind in old age due to a optic nerve atrophy. The patient vaguely remembered that he might have had squint surgery in childhood but had no documentation.**	
	**Summaries from initial and follow-up visits**	**Interventions and therapy**
Mid-January 2017	VA loss on the left eye (LE)	
February 2017 (5 months after the on-set of the right eye)	Hospitalization at Clinic due to VA loss on the previously healthy left eye. VA RLE counting fingers on 2 meters, Color vision RLE 1/15, central scotoma bilaterally. On fundoscopy the optic discs were bilaterally hyperemic with tortuous blood vessels. Edematous pRNFL and thinning of the inner retina in the macular region. FA: no leakage,	Systemic corticosteroid therapy for 3 days Solumedrol i.v. (1g/day), no improvement. MRI of the head and brain, no signs of demyelination, aqp4 negative, antiMOG negative, excluded all other possible signs of the optic atrophy (infectious, paraneoplastic, compressive etc.)

		Blood taken for genetic testing.
October 2017 (8 months after the onset)	VA RLE hand movement Color vision RLE 1/15, LE 1/15, central scotoma bilaterally. On fundoscopy both optic discs pale, thinning of the inner retina on OCT bilaterally, further progression of the pRNFL thinning EF: P 50 amplitude normal, lower on amblyopic eye, decreased N95 wave more on the amblyopic eye, N95/P50 ratio RE 0.76 LE:0.89, VEP undetectable	Genetic testing for 3 common mutations revealed common mutation. Patient started with Idebenone
January 2018 (12 months after the onset on the left eye)	VA RLE light perception Color vision RLE 0/15, small remanent of the visual field. On fundoscopy both optic discs pale, thinning of the inner retina on OCT bilaterally, significant pRNFL thinning.	Patient left the country and was lost for further follow-up

Table S5. Timeline of the disease progression in Patient 3.

Date	Relevant past medical history	
	Low vision in the left eye since childhood due to esodeviation which was never operated on. History of arterial hypertension. Two relatives on the mother's side in the family had a similar episode of vision loss and then improvement at a younger age. Unfortunately, these relatives live in another country and were not available for screening and genetic testing.	
	**Summaries from initial and follow-up visits**	**Interventions and therapy**
June 2010	VA loss on the previously healthy right eye	
June 2010 (2-3 weeks after the onset of the right eye)	Hospitalization at Clinic. VA RE counting fingers on 3 meters, LE: counting fingers at 2,5 m Color vision RLE 0/15. VF Bilateral visual field constriction of 15-20 deg, enlarged right blind spot and excluded left blind spot. On fundoscopy right optic disc was pink with sharp borders, left slightly paler temporally FA: no leakage, EF: P 50 amplitude was reduced, lower on amblyopic eye, The N95 was decreased, agin more on the amblyopic eye decreased N95 wave more on the amblyopic, N95/P50 ratio RE 1.52 LE:1.27, delayed and decreased VEP P100 more on the amblyopic eye.	Systemic corticosteroid therapy for 3 days Solumedrol i.v. (1g/day), no improvement. MRI of the head and brain, no signs of demyelination, aqp4 negative, antiMOG negative, excluded all other possible signs of theoptic atrophy (infectious, paraneoplastic, compressive etc)
October 2010 (4 months after the onset)	VA RLE 0,1 Color vision RLE 1/15, VF REscotoma in lower half of visual field LE scotoma on nasal visual field. On fundoscopy both optic discs were still pink. Decreased sensitivity and extentric fixation on microperimetry.	Bilateral amblyopia suspected due to the absence of the optic disc pallor
June 2011 (12 months after the onset)	VA RLE 0,1 Color vision RLE 1/15, central scotoma bilaterally. On fundoscopy temporal pallor of the optic discs started to show, thinning of the inner retina on OCT bilaterally and thinning of the pRNFL on the RE T ans TS, LE temporal half of the optic disc.	Hereditary optic neuropathy was suspected, and blood taken for genetic analysis
December 2011 (1.5 years after the onset)	Slight VA improvement RE 0.2 LE:0.1 Color vision RE 0/15, LE 1/15, smaller scotomas in visual field bilaterally. On fundoscopy both optic discs pale, thinning of the inner retina on OCT bilaterally, further progression of the pRNFL thinning.	Gentic testing for 3 common mutations revealed presence of the common mutation
June 2012 (2 years after the onset)	Significant VA improvement RE 0.8, LE 0.1 Color vision RE 3/15, LE 1/15, significant reduction of the visual field bilaterally. On fundoscopy both optic discs paler, thinning of the inner retina on OCT bilaterally, further progression of the pRNFL thinning, with still preserved nasal segment on both eyes. EF: P 50 amplitude improved, but was still lower on amblyopic eye, The N95 was decreased on the amblyopic eye, normal on the LHON but in the level of the baseline, N95/P50 ratio RE 0.83 LE:0.74, delayed (more on amblyopic) and decreased (more on LHON) VEP P100 wave.	
March 2018 (8 years after the disease onset)	Complete VA recovery on the RE 1.0, LE 0.1 Color vision RE 6/15, LE 1/15, further reduction of the visual field scotomas bilaterally. On fundoscopy both optic discs were paler, with thinning of the inner retina on OCT bilaterally in the macular region, slight progression of the pRNFL thinning in the TI segment on LHON eye, and no progression in other segments in comparison to 2012. EF: P 50 amplitude normal on	

	both eyes, still lower on amblyopic, The N95 was borderline decreased on the amblyopic eye, normal on the LHON but in the level of the baseline, N95/P50 ratio RE 1.04 LE:1.05, borderline delayed and still significantly decreased VEP P100 wave (more on the amblyopic eye). Microperimetry showed significant improvement in sensitivity.
